# Development of hybrid materials based on sponge supported reduced graphene oxide and transition metal hydroxides for hybrid energy storage devices

**DOI:** 10.1038/srep07349

**Published:** 2014-12-08

**Authors:** Deepak P. Dubal, Rudolf Holze, Pedro Gomez-Romero

**Affiliations:** 1 Catalan Institute of Nanoscience and Nanotechnology, CIN2, ICN2 (CSIC-ICN), Campus UAB, E-08193 Bellaterra, Barcelona, Spain; 2 Technische Universität Chemnitz, Institut für Chemie, AG Elektrochemie, D-09107 Chemnitz, Germany; 3 Consejo Superior de Investigaciones Científicas (CSIC), Spain

## Abstract

Earnest efforts have been taken to design hybrid energy storage devices using hybrid electrodes based on capacitive (rGO) and pseudocapacitive (Ni(OH)_2_ and Co(OH)_2_) materials deposited on the skeleton of 3D macroporous (indicate sponge material) sponge support. Conducting framework was formed by coating rGO on macroporous sponge on which subsequent deposition of Ni(OH)_2_ and Co(OH)_2_ was carried out. The synergetic combination of rGO and Ni(OH)_2_ or Co(OH)_2_) provides dual charge-storing mechanisms whereas 3D framework of sponge allows excellent accessibility of electrolyte to hybrid electrodes. Moreover, to further increase the energy density, hybrid devices have been fabricated with SP@rGO@Ni or SP@rGO@Co and SP@rGO as positive and negative electrodes, respectively. These hybrid devices operate with extended operating voltage windows and achieve remarkable electrochemical supercapacitive properties which make them truly promising energy storage devices for commercial production.

Nowadays, energy-related science and technology are benefiting from a big tsunami of hybridization, starting from hybrid materials to hybrid devices^1^. In hybrid materials, there are synergistic effects of materials properties whereas hybrid devices combine the different mechanisms of devices in a single one. The field of hybrid energy storage devices is no exception. Among different energy storage systems playing on the chessboard of Ragone plot, supercapacitors are getting increasing attention from scientists and industrialists alike due to their excellent performance at high power rates^2^,^3^. Indeed, supercapacitors are intrinsically well suited to provide high power density by storing charge non-faradically at the electrode/electrolyte interface (electrochemical double layer capacitor, EDLC) or through redox mechanism at the electrode surface (pseudocapacitor) hence allowing for high power, fast charge-discharge rates and excellent cycling life^4^. As we look back, supercapacitors are highly effective and are integrated in many of our daily-live applications from portable devices such as mobile-phones, laptops, cameras to hybrid electric vehicles etc. Despite this success these systems still have boundaries such as low energy density in EDLC, or short cycle life and limited power capability in pseudocapacitors^5^. Looking at both systems, the demerits of one type are the strengths of the other and vice-versa. Thus, the hybridization of EDLC and pseudocapacitor materials is an appealing path to solve the problems related to both systems^6^.

There are two kinds of hybrids; one is the “hybrid electrode” in which pseudocapacitive/faradic and non-faradic materials are combined in single electrode in order to take the advantage of both charge storing mechanisms^7^, whereas the second is the “hybrid device or hybrid combination” in which a pseudocapacitive/faradic electrode and non-faradic one are combined in a single cell^8^. The most promising approach to develop hybrid electrodes is to integrate non-faradaic (carbon based) and pseudocapacitive/faradic materials (transition metal oxides/hydroxides, polyoxometalates, conducting polymers and Li-intercalates). Simple mixing of two different materials will not be an effective mode to fabricate hybrid electrodes due to difficulties in interfacial contacts between them. The proper hybridization of components through rational design with control over morphology, size and uniformity of active material, as well as facile processing are key parameters to obtain inexpensive yet high-performance hybrid electrodes^9^. An emerging new concept to fabricate hybrid electrodes is designing carbon supported substrates with pseudocapacitive materials under the control of their morphologies and sizes such as carbon nanofiber textile, carbon nanotube/graphene coated textile, and three dimensional macroporous graphene frameworks^10^,^11^,^12^,^13^. To this list of possible substrates for hybrid supercapacitive electrodes, a new very practical contender has been recently added, namely, commercially available sponges with 3D porous structure coated with carbon. The sponge substrate provides smooth inner and outer sides of the skeleton for the deposition of materials and its soft, lightweight, flexible and cost-effective nature makes it more attractive for flexible devices. Moreover, macroporosity and high surface area of sponge provide an easy path to the electrolyte to reach the entire surface of the electrodes where redox reactions can take place.

In the long list of possible chemical methods, the chemical bath deposition (CBD) method is best suited to prepare large-scale thin coatings of different materials with diverse and controllable morphologies at low temperature and low cost on any type of substrate. Moreover, direct growth of material by CBD method enhances the interfacial contacts and avoids the use of unnecessary binders and additives.

In this work we have successfully used both of those possible optimization pathways (CBD on foam substrates) to improve the performance of supercapacitors in aqueous system. According to the equation for energy storage i.e. E = 0.5 CV^2^, the performance of the supercapacitors can be enhanced by increasing capacitance and extending the operational voltage window using different configurations. The work we present here deals with:

1. Construction of unique hybrid electrodes to improve capacitance: Using macroporous 3D sponge (SP) substrate coated with reduced graphene oxide (rGO), we have deposited two different materials Ni(OH)_2_ and Co(OH)_2_ by inexpensive CBD method to fabricate open porous, high surface area hybrid electrodes to achieve synergistic effects of EDLC and pseudocapacitive materials at low cost.
2. Fabrication of hybrid device to extend working potential window: The hybrid device is configured by using hybrid electrode (above mentioned) as positive with rGO coated sponge as negative electrodes in single cell to extend working potential window.

Thus, we have presented an integrated approach from hybrid materials to hybrid devices. These hybrid materials effectively increase the capacitance and hybrid configurations extend the working potential windows of device in aqueous system which consequently enhances the supercapacitive properties of the devices.

## Results

**Figure 1:**
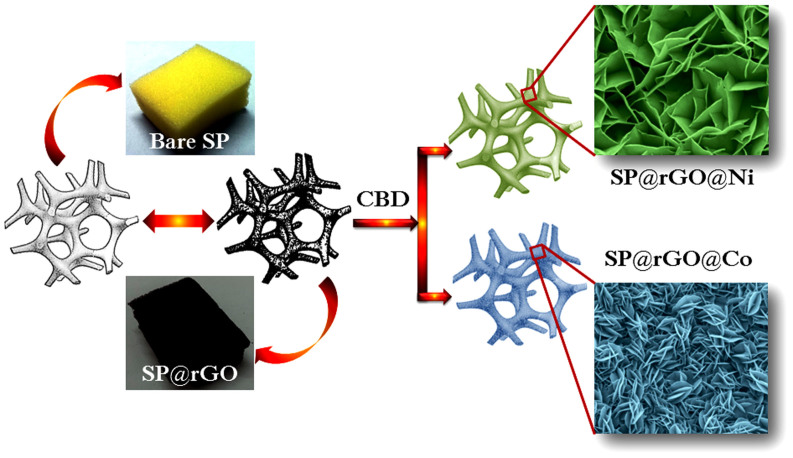
Schematic representation with digital photographs of fabrication of hybrid materials based on rGO and transition metal hydroxides (Ni(OH)_2_ and Co(OH)_2_) onto skeleton of 3D macroporous sponge.

Synthesis of SP@rGO@Ni and SP@rGO@Co hybrid electrodes involves two steps: first, coating of rGO onto the sponge substrate by “dip and dry” method and subsequently, depositions of nanostructured Ni(OH)_2_ and Co(OH)_2_ carried out by CBD method (see Fig. 1). The bare sponge was first coated with rGO nanosheets via dipping into rGO ink in aqueous solution and subsequent drying in an oven. By this way, 3D macroporous conducting framework with high surface area was formed which provides enough space for the heavy and uniform deposition of nanostructured Ni(OH)_2_ and Co(OH)_2_ onto the skeleton of the sponge easily. Sponge surface was uniformly covered with aggregates of rGO sheets (see Supporting information S2) with almost completely transparent and crumpled to a curly, wavy shape.

**Figure 2:**
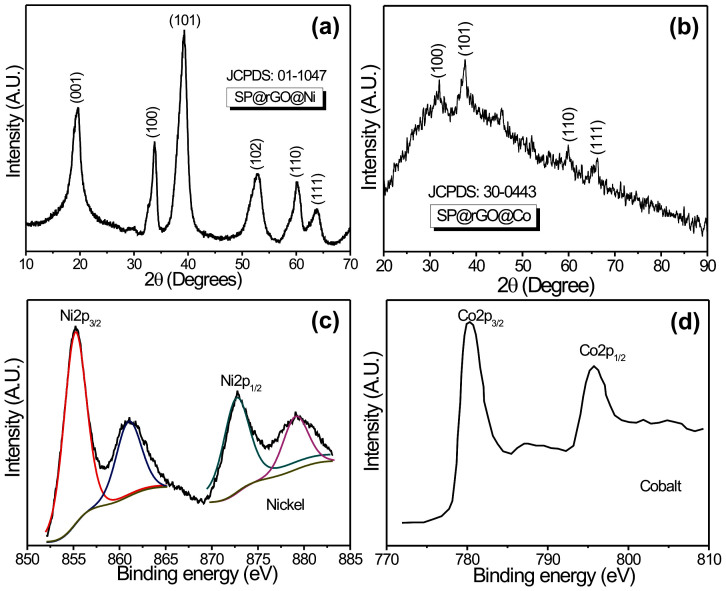
SEM images of (a), (c) and (e) SP@rGO@Ni and (b), (d) and (f) SP@rGO@Co hybrid at two different magnifications with corresponding cross-sectional images showing a thick layer of Ni(OH)_2_ and Co(OH)_2_ coated on SP@rGO support.

SEM images as shown in Fig. 2(a, b) reveal, the 3D open porous nature of the sponge substrate is maintained even after the coating of rGO and deposition of Ni(OH)_2_ and Co(OH)_2_ nanostructures. Fig. 2(c and d) shows hierarchically porous well networked structure of thin β-Ni(OH)_2_ nanosheets and β-Co(OH)_2_ nanoflakes. These nanostructures are interconnected with each other, providing open porous structure which enables the full exposure of the arrays to the electrolyte. The open geometry between the arrays allows easier electrolyte penetration into the inner region of the electrode, increasing the utilization of the active materials. The skeleton of the sponge is fully covered by both Ni(OH)_2_ and Co(OH)_2_ nanostructures so that no rGO is exposed to the surface (Supporting information S3). The thicknesses of the Ni(OH)_2_ and Co(OH)_2_ coatings were found to be ~1.2 and ~1.3 μm, respectively which can be measured by the cross-section SEM images in Fig. 2(e–f). A possible growth process will be as follows: Initially, dissolved ammonia forms a complex with metal ions (Ni^2+^ and Co^2+^), which decreases the free metal ion concentration and reduces the rate of crystal growth. Formation of metal ammine complex avoids homogeneous nucleation in the solution due to the binding of metal ions and provides priority to heterogeneous nucleation on the substrate. Further, as the reaction time increases (at a constant temperature), hydrolysis of HMT molecules takes place. This increases the concentration of OH^−^ in the solution which encourages the formation of thin Ni(OH)_2_ nanosheets and Co(OH)_2_ nanoflakes^15^. Here, HMT provides controlled supply of ammonia via the hydrolysis reaction, while unreacted HMT selectively adsorbs on the metal hydroxide nanoparticles due to its high binding capacity^16^, resulting in the formation of nanosheets/nanoflakes. According to thermodynamics, the surface energy of an individual nanosheet or nanoflake is high therefore they aggregate perpendicularly to the surface planes in order to decrease the surface energy^17^. Therefore, as the reaction proceeds, the thin sheets and flakes would self-aggregate to form interconnected Ni(OH)_2_ nanosheets and Co(OH)_2_ nanoflakes clusters for minimizing the overall surface energy.

**Figure 3:**
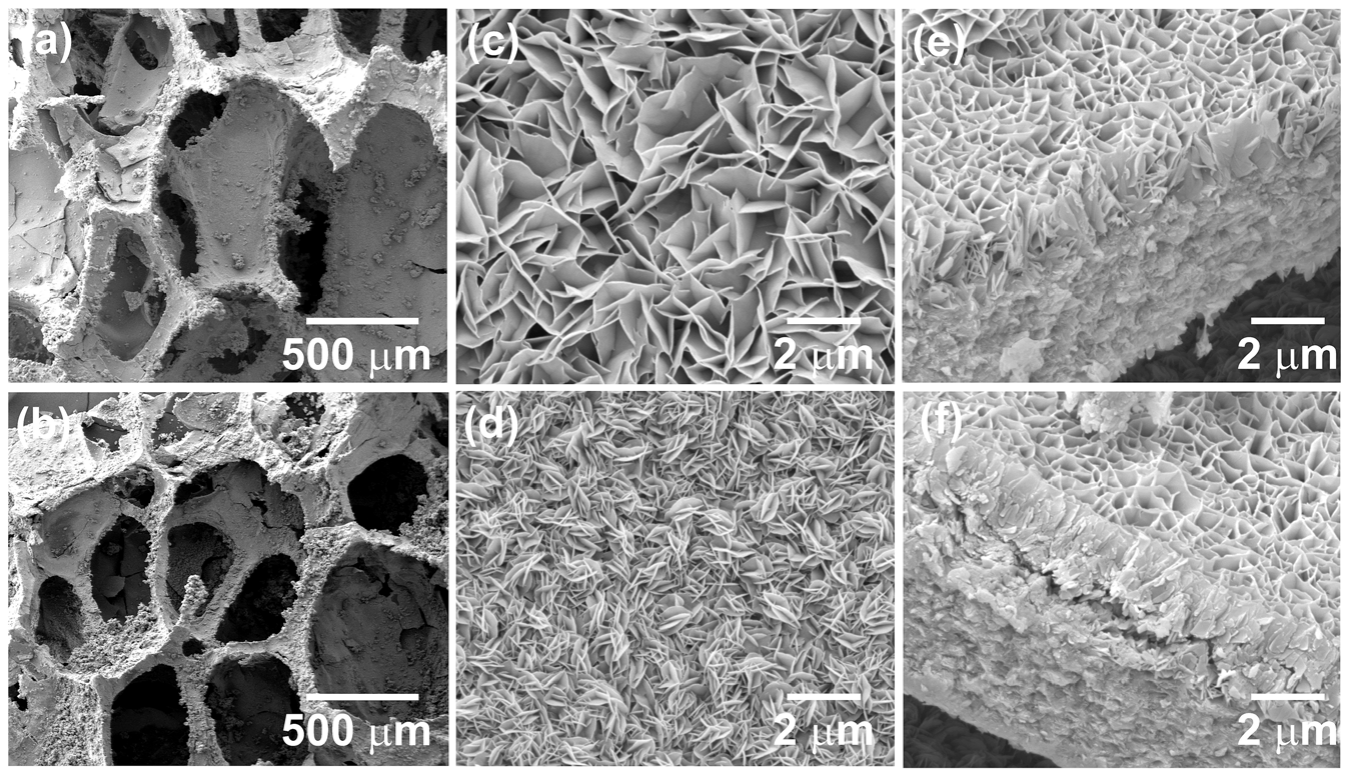
(a), (b) XRD patterns and (c), (d) core level XPS of Ni2p and Co2p spectra for SP@rGO@Ni and SP@rGO@Co hybrid materials, respectively.

Fig. 3(a, b) shows the XRD patterns of SP@rGO@Ni and SP@rGO@Co, respectively. As seen from the XRD patterns, it is evidenced that both materials are polycrystalline in nature. The diffraction peaks (001), (100), (101), (102), (110) and (111) in Fig. 3(a) are readily indexed to a pure single phase of β-Ni(OH)_2_ with the hexagonal brucite structure [JCPDS 14-0117] whereas the peaks (100), (101), (110) and (111) correspond to pure hexagonal β-Co(OH)_2_ according to JCPDS 30-0443 (Fig. 3(b)). Moreover, broadened diffraction peaks evidence that both deposited materials are nanocystalline in nature. Due to the small content of rGO coated on the sponge substrate, the corresponding peak for rGO was not detected in XRD. Further, to investigate the oxidation states of elements, XPS analysis was carried out. The XPS Ni2p peak (Fig. 3(c)) consisted of two spin-orbit doublets characteristic of Ni^2+^ and Ni^3+^ and two shake-up satellites. The binding energy of Ni2p_3/2_ was about 855.2 eV, which is consistent with the previously reported value^18^. The XPS Co2p peak (Fig. 3(d)) consisted of two spin-orbit doublets characteristic of Co^2+^ and Co^3+^ and two shake-up satellites. The binding energy corresponding to Co2p_3/2_ was about 780.2 eV^18^.

**Figure 4:**
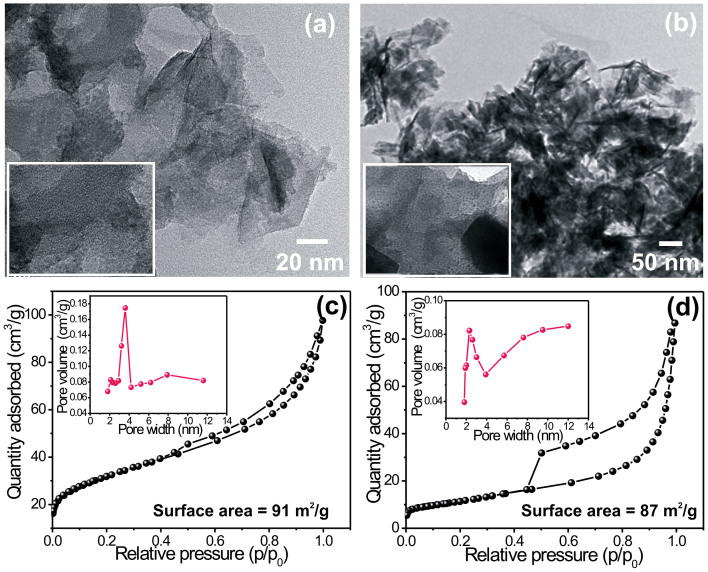
(a), (b) TEM images of Ni(OH)_2_ and Co(OH)_2_ samples with corresponding high magnified TEM images (inset), respectively, (c), (d) Nitrogen adsorption/desorption isotherm of Ni(OH)_2_ and Co(OH)_2_ samples and inset shows the corresponding BJH pore size distribution plots.

TEM analysis is further carried out to provide more insight into the detailed microstructures of the Ni(OH)_2_ and Co(OH)_2_ samples. TEM image shown in Fig. 4(a) presents a panoramic view of thin nanosheets of Ni(OH)_2_. It is further seen that, the nanosheets are loosely packed and have separate existence. Fig. 4(b) shows the TEM image of a representative flowerlike Co(OH)_2_ nanostructure which is in good agreement with the results from the FESEM image. In addition to this the edge of the hierarchical Ni(OH)_2_ nanosheets and Co(OH)_2_ nanoflakes further indicates the ultrathin nature of a single component with almost transparent feature. Fig. 4(c–d) shows the nitrogen adsorption and desorption isotherms and the corresponding pore size distributions (inset) of Ni(OH)_2_ and Co(OH)_2_ samples, respectively. The specific surface areas were calculated from the Brunauer-Emmett-Teller (BET) method and the pore size distributions (PSD) were obtained by means of the Barrett-Joyner-Halenda (BJH) equation using the adsorption isotherm branch. The BET specific surface areas of Ni(OH)_2_ and Co(OH)_2_ samples were found to be 91 and 87 m^2^/g, respectively. The pore size distribution curves of Ni(OH)_2_ and Co(OH)_2_ samples are also shown in inset of Fig. 4(c–d). The most of the pores are observed in the range of 2–10 nm, with a centered pore distributions at around 3.57 nm, and 2.29 nm for Ni(OH)_2_ and Co(OH)_2_ samples, respectively indicating that pores of hierarchical nanosheets and nanoflakes are mainly mesopores with some micropores. This mesoporous structure of Ni(OH)_2_ and Co(OH)_2_ could provide easy access for the ions in the electrolyte as well as short diffusion path for intercalation and de-intercalation. The porosity of samples is an amalgamation of internal space of the agglomerated nanosheets/nanoflakes and the porosity due to internal space of bunch of nanosheets/nanoflakes. The pore size distribution of Ni(OH)_2_ and Co(OH)_2_ samples is slightly narrow which further supports the better homogeneity in the surface morphology and porosity of samples. These surface properties directly affect the electrochemical properties of electrode materials.

**Figure 5:**
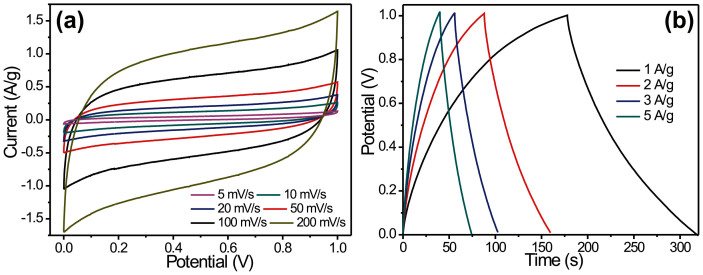
(a) Cyclic voltammetry curves of SP@rGO electrodes collected at different scanning rates in 2 M KOH electrolyte, respectively (b) Galvanostatic charge/discharge curves of SP@rGO electrodes at different current densities.

To evaluate the electrochemical performance SP@rGO electrodes, a typical two electrode configuration was constructed. Two pieces of SP@rGO sponge as electrodes, one piece of separator and 2 M KOH as electrolyte was used. The sponges based conductive framework with high accessible surface area and macroporous open pore structures were acted as electrode as well as current collector which also provides highly porous channels for the full access of electrolytes to active materials. The cyclic voltammetry (CV) curves (Fig. 5(a)) indicate that the SP@rGO devices can be operated over a wide range of scan rates: from 5 up to 500 mV/s. Even at the scan rate of 500 mV/s, the cyclic voltammograms retain the rectangular shape showing the ideal electrochemical double layer behavior of rGO coated sponge electrode. The high scan rate that SP@RGO can achieve implies that ultrahigh power density can be obtained in this device. The galvanostatic charge/discharge curve at a specific current of 5 A/g (Fig. 5(b)) shows an ultrafast charge discharge rate, linear dependence on voltage and time, and very small voltage drop demonstrating that the SP@rGO electrodes can work at high specific current. Thus, the results demonstrate that SP@rGO electrode exhibit high EDLC performance and would be a promising energy storage substrate.

**Figure 6:**
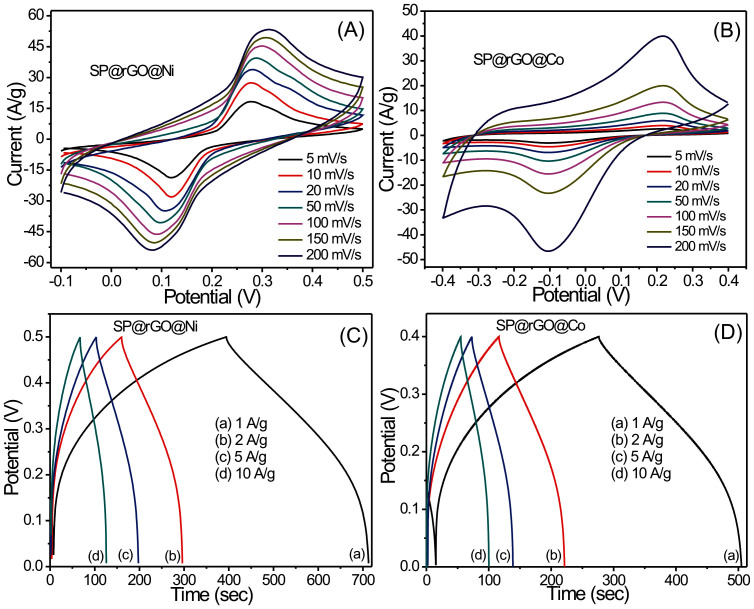
(A), (B) Cyclic voltammetry curves of SP@rGO@Ni and SP@rGO@Co hybrid electrodes collected at different scanning rates in 2 M KOH electrolyte, respectively (C), (D) Galvanostatic charge/discharge curves of SP@rGO@Ni and SP@rGO@Co hybrid electrodes at different current densities, respectively.

To demonstrate the advantages of hybrid electrodes (SP@rGO@Ni and SP@rGO@Co) which are obtained by hybridization of pseudocapacitive and non-faradic materials, we have investigated the electrochemical properties in two electrode configuration devices. The SP@rGO@Ni and SP@rGO@Co served as electrodes as well as current collectors with binder-free, highly porous and conductive framework which provides full access to electrolyte ions. The shapes of the CV curves of SP@rGO@Ni and SP@rGO@Co symmetric devices (Fig. 6(A, B)) indicate that the capacitance characteristic is different from that of electric double layer capacitance in which the shape is normally close to an ideal rectangular shape. Each CV curve consists of a pair of strong redox peaks, which indicates that the capacitance characteristics are mainly governed by redox reactions. It must also be noted that as the scan rate increased, the potential of the anodic and cathodic peaks shift to more positive and negative directions, respectively. The possible charge storing mechanisms for Ni(OH)_2_ and Co(OH)_2_ are as follows^19^: 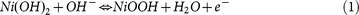

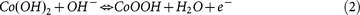
 As expected, there is an increase in current with the scan rate while the CV curves retain the shape when the applied scan rate is very high (200 mV/s). This indicates a proper hybridization of (Ni(OH)_2_ or Co(OH)_2_) with rGO which allows efficient ion transport at the interface of hybrid electrodes and electrolyte. Moreover, both outer- and the inner-pore surfaces of the hybrid electrode materials are effectively utilized for the intercalation^20^. The highest specific capacitance values obtained for symmetric SP@rGO@Ni and SP@rGO@Co devices were 812 F/g (1.7 F/cm^2^) for mass loading of 4.2 mg/cm^2^ whereas 752 F/g (1.46 F/cm^2^) for mass loading of 3.9 mg/cm^2^ at a potential scan rate of 5 mV/s, respectively. The galvanostatic charge-discharge curves of both hybrid symmetric devices are shown in Fig. 6(C–D) at different current densities. The curves show that these hybrid systems can be operated at a wide range of current densities with small voltage drop, further demonstrating the excellent electrochemical performance of SP@rGO@Ni and SP@rGO@Co nanostructured hybrid electrodes. The charge-discharge curves reflect the faradic behavior of the Ni(OH)_2_ and Co(OH)_2_ samples. It should also be noted that the small voltage-drop at the start of discharge indicates a low internal resistance, excellent conductivity, and the capability for a quick charge. This may be attributed to the inclusion of rGO in Ni(OH)_2_ and Co(OH)_2_ nanostructures. The estimated specific capacitance values of SP@rGO@Ni and SP@rGO@Co hybrid electrodes were found to be 806 and 747 F/g at a current density of 2 A/g. The good capacitive behavior of SP@rGO@Ni and SP@rGO@Co electrodes is due to the combined contribution of rGO with Ni(OH)_2_ and Co(OH)_2_ nanostructures which provides large internal accessible surface sample matrix, decrease the ion diffusion resistance and enhance the electro-active surface utilization during the redox process. Moreover, the 3D macroporous framework of the sponge and numerous nano-channels in Ni(OH)_2_ nanosheets and Co(OH)_2_ nanoflakes act as “ion-buffering reservoirs” which reduce the mean free path of the ions and facilitate faster ionic and electronic kinetics, thus maximizing the reversible insertion/de-insertion reactions^21^.

**Figure 7:**
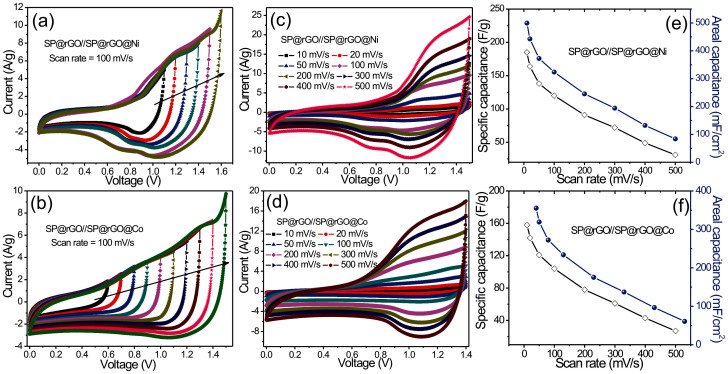
CV curves of the (a) SP@rGO//SP@rGO@Ni and (b) SP@rGO//SP@rGO@Co hybrid devices at different working voltages at a scan rate of 100 mV/s. CV curves of (c) SP@rGO//SP@rGO@Ni and (d) SP@rGO//SP@rGO@Co hybrid devices at different scan rates. Variation of specific and areal capacitances of (e) SP@rGO//SP@rGO@Ni and (f) SP@rGO//SP@rGO@Co devices as function of the scan rate.

In order to further increase the specific energy without sacrificing power delivery and cycle life in the aqueous electrolyte; supercapacitors have been proposed to reach a higher cell voltage through the asymmetric mode where one electrode is made of a faradaic material and the other of a non-faradaic one. Several reports are available on this approach^22^,^23^. Here, we are going to demonstrate somewhat different hybrid design which is comprised of one non-faradic electrode (SP@rGO) with other hybrid electrode which combines non-faradic and pseudocapacitive characteristics (SP@rGO@Ni or SP@rGO@Co) in aqueous system. Due to the high hydrogen evolution overpotential of rGO in aqueous media^24^ and the excellent capacitive features of SP@rGO@Ni and SP@rGO@Co synthesized in this work, hybrid configuration is tested for achieving the goal of high-energy and high-power densities. In this hybrid device, charges are stored non-faradically in rGO electrodes, and through redox reactions in the hybrid metal hydroxide electrodes. The specific capacitances of the SP@rGO@Ni, SP@rGO@Co and SP@rGO electrode were calculated to be 812 F/g (4.2 mg/cm^2^), 752 F/g (3.9 mg/cm^2^) and 127 F/g (1.9 mg/cm^2^) at the scan rates of 5 mV/s, respectively. Note that in order to reach the highest cell voltage, the charges stored in both electrodes must be balanced by adjusting the mass loading of the electrodes. The positive to negative electrode mass ratio was calculated by the following equation to achieve a charge balance q^+^ = q^−^.


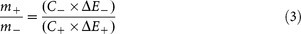
where q^+^, q^−^, m_+_, m_−_, C_+_, C_−_, E_+_, and E_−_ are the charge, mass, specific capacitance and potential windows for the cathode (+) and anode (−). Hence, the mass ratio between the positive and negative electrodes of the SP@rGO//SP@rGO@Ni and SP@rGO//SP@rGO@Co hybrid devices should be 3.19 and 2.37, respectively. Accordingly, the charge balance between positive and negative electrodes, the optimal positive-to-negative mass ratio was determined to be 4.12 mg/cm^2^:1.28 mg/cm^2^ and 3.23 mg/cm^2^:1.33 mg/cm^2^ for SP@rGO//SP@rGO@Ni and SP@rGO//SP@rGO@Co hybrid devices, respectively. Fig. 7(a–b) show the CV curves at different voltage windows for SP@rGO//SP@rGO@Ni and SP@rGO//SP@rGO@Co hybrid devices respectively at a scan rate of 100 mV/s. It can be clearly seen that the fabricated hybrid devices show a good capacitive behavior and expand their potential windows up to 1.5 and 1.4 V for SP@rGO//SP@rGO@Ni and SP@rGO//SP@rGO@Co cells, respectively. Further increase in voltage window causes some irreversible reaction since sudden increase in current has been observed (see Fig. 7(a–b)). It was further found that the current and consequently the specific capacitance greatly increase as the operating voltage window is increased, which indicates the improvement in stored energy and delivered power. As a consequence, the overall performance of the supercapacitor could also be remarkably improved. As is generally known, operating at higher voltage will be favorable for reducing the number of devices in series required to reach the desired output voltage in practical applications. Thus, we chose an operation voltage window of 1.5 V and 1.4 V for SP@rGO//SP@rGO@Ni and SP@rGO//SP@rGO@Co cells in 2 M KOH aqueous electrolyte to further investigate the electrochemical performance, respectively. Fig. 7(c–d) shows the CV curves of the SP@rGO//SP@rGO@Ni and SP@rGO//SP@rGO@Co hybrid devices measured at various scan rates of 10–500 mV/s. These CV curves exhibit a non-rectangular current response on voltage reversal with small redox peaks. The shapes of CV curves remain unchanged even at a high scan rate of 500 mV/s, suggesting that the cell possesses excellent rate capability which is desirable for high-power supercapcitors. The specific capacitances of hybrid devices (based on the total mass of the active materials of the two electrodes) at different scan rates calculated from the CV curves is presented in Fig. 7(e–f). The highest gravimetric capacitance (areal capacitance) for SP@rGO//SP@rGO@Ni and SP@rGO//SP@rGO@Co hybrid devices were found to be 185 (0.49 F/cm^2^) and 158 (0.35 F/cm^2^) at 10 mV/s, which are greatly larger than the values reported for hydroxide based asymmetric supercapacitors^25^,^26^,^27^,^28^. The specific capacitances decrease gradually with increasing scan rate as diffusion limits the movement of electrolyte ions at high scan rates because of the time constraint and only the outer active surface can be utilized for charge storage, resulting in a lower electrochemical utilization of electroactive materials^29^. The higher specific capacitance could be obtained at higher operation voltage windows thanks to the redox reactions of Ni(OH)_2_ and Co(OH)_2_. Importantly, it should be pointed out that the specific capacitance is calculated based on the total mass of the active material on both electrodes. The excellent performance of the hybrid devices can thus be attributed to the high capacitance and rate performance as well as the synergistic effects of both the SP@rGO and SP@rGO@Ni/SP@rGO@Co hybrid electrodes.

**Figure 8:**
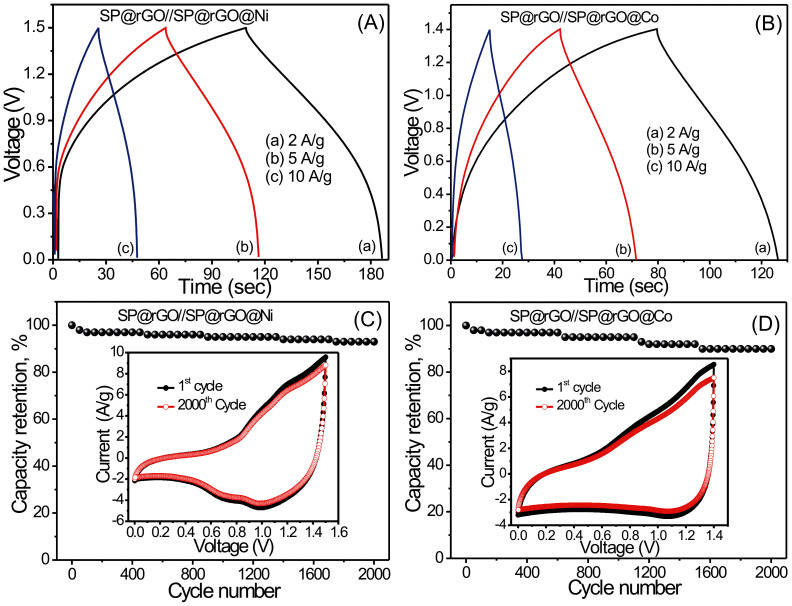
Galvanostatic charge-discharge curves of (A) SP@rGO//SP@rGO@Ni and (B) SP@rGO//SP@rGO@Co hybrid devices at different current densities. Variation of capacity retention of (C) SP@rGO//SP@rGO@Ni and (D) SP@rGO//SP@rGO@Co hybrid devices with number of cycles at 100 mV/s scan rate along with corresponding CV curves (inset) at 1^st^ and 2000^th^ cycles.

Galvanostatic charge/discharge curves of hybrid cells were recorded with various current densities to further evaluate the electrochemical performance as shown in Fig. 8(A–B). It can be seen that all of the curves are not ideal straight lines, indicating the involvement of a redox reaction process. Furthermore, there is an initial drop in potential, which may be caused by the internal resistance as well as different rates of oxidation and reduction reactions. Very small voltage-drop is observed at initial discharge curve indicating excellent conductivity and fast I–V response.

Finally, a long cycling life is an important requirement for supercapacitor applications. Long cycling-life tests were carried out for the SP@rGO//SP@rGO@Ni and SP@rGO//SP@rGO@Co hybrids by repeating the CV test at a scan rate of 100 mV/s for 2000 cycles (see inset of Fig. 8(C–D)). Fig. 8(C–D) shows the capacitance retention ratio of the hybrid devices as a function of the cycle number. It is worth noting that the specific capacitance decreases suddenly after the initial 50 cycles, which is probably related to pulverization and loss of electrical contact between the active material and the current as well as wettability issues. After 2000 cycles, the SP@rGO//SP@rGO@Ni and SP@rGO//SP@rGO@Co hybrid devices display an excellent long cycle life with 90% and 87% retention of their initial specific capacitance, respectively, demonstrating superior long-term electrochemical stability. Moreover, excellent stability confirms the proper hybridization of two different (pseudocapacitive and non-faradic) materials. Such a stable and connecting structure helps to alleviate the structure damage caused by volume expansion during cycling process, resulting in an enhanced stability. Thus, such a cycling performance is highly competitive with those of some other asymmetric supercapacitors, such as Ni(OH)_2_//activated carbon (82% retention after 1000 cycles)^30^, LiNi_1/3_Co_1/3_Mn_1/3_O_2_//AC (ca. 80% retention after 1000 cycles)^31^, graphene-MnO_2_//graphene (79% retention after 1000 cycles)^32^, and porous NiO//carbon devices (50% after 1000 cycles)^33^.

**Figure 9:**
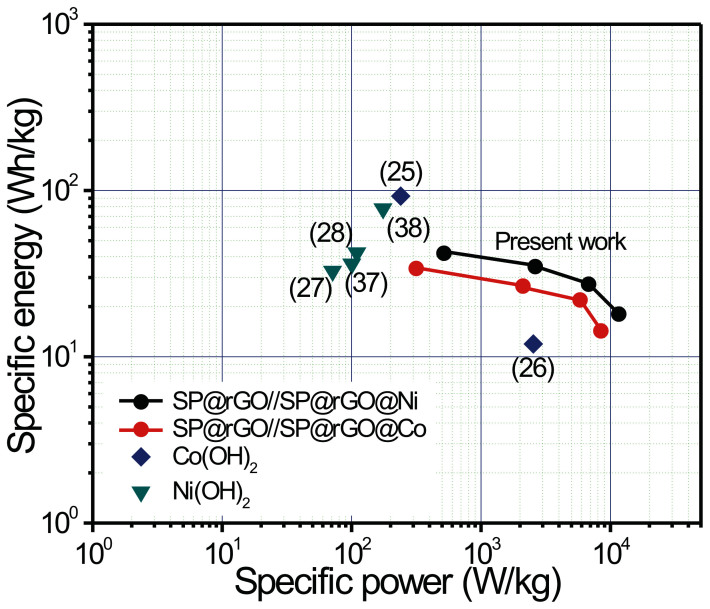
The power density versus energy density of SP@rGO//SP@rGO@Ni and SP@rGO//SP@rGO@Co hybrid devices in a Ragone plot.

Energy density and power density are two key factors for evaluating the performance of supercapacitors. A high performance supercapacitor is expected to provide high energy density or high specific capacitance at high discharge rates. Fig. 9 presents the Ragone plot of the SP@rGO//SP@rGO@Ni and SP@rGO//SP@rGO@Co hybrid devices. The as-assembled SP@rGO//SP@rGO@Ni hybrid device with a cell voltage of 1.5 V can deliver a high specific energy of 42.02 Wh/kg with maximum specific power of 11 kW/kg whereas SP@rGO//SP@rGO@Co cell exhibits 33.01 Wh/kg with specific power of 8 kW/kg. Furthermore, these devices can retain an energy density >10 Wh/kg even at a high power density. The energy density demonstrated in this work is significantly higher than those obtained for other carbon-based symmetric capacitors in aqueous electrolytes, such as activated carbon (<10 Wh/kg)^34^, carbon nanotubes (<10 Wh/kg)^35^, graphene (9.1 Wh/kg)^36^. The excellent performance is also comparable or superior to other asymmetric cells with mild aqueous electrolytes, such as Co(OH)_2_//GO (11.94 Wh/kg)^25^, Co(OH)_2_//AC (92.7 Wh/kg)^26^, Ni(OH)_2_//AC (32.7 Wh/kg)^27^, β-Ni(OH)_2_//AC (36.2 Wh/kg)^37^, Ni(OH)_2_-graphene//porous graphene (77.8 Wh/kg)^38^, α-Ni(OH)_2_//AC (42.3 Wh/kg)^28^, Ni(OH)_2_//AC (35.7 Wh/kg)^39^, Ni(OH)_2_/ultrathin graphite foam//activated microwave exfoliated graphite oxide (13.4 Wh/kg)^40^.

## Discussion

**Figure 10:**
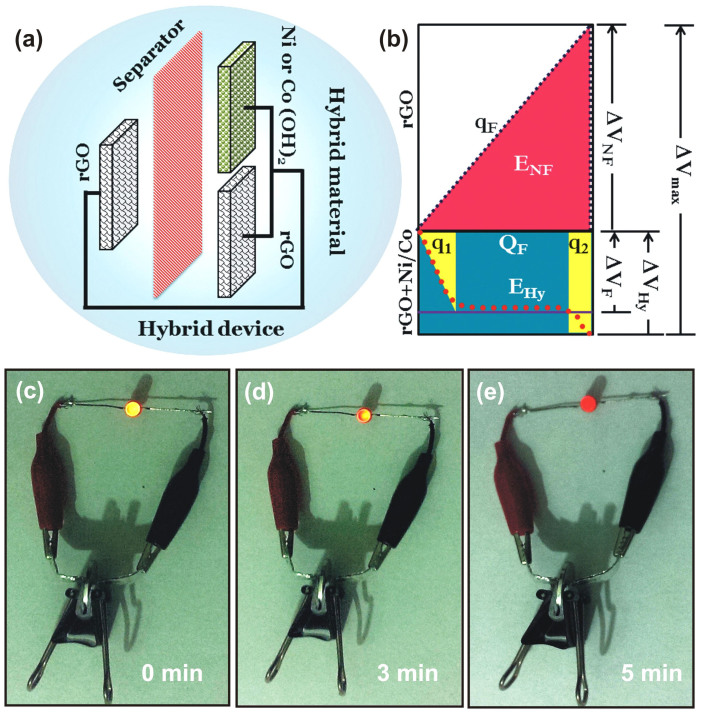
(a), (b) Schematic representation of hybrid combination (device) of hybrid electrode (SP@rGO@Ni or SP@rGO@Co) and SP@rGO electrode with propylene carbonate paper as separator along with charge-potential profile. (c)–(e) Demonstration of the use of the SP@rGO//SP@rGO@Ni hybrid device is given with a light emitting diode (LED).

For a better understanding of the working of hybrid device with hybrid electrode, the schematic of hybrid device is sketched in Fig. 10(a). The characteristic of hybrid electrode is hybridization of pseudocapacitive component (Ni(OH)_2_ and Co(OH)_2_) and non-faradic component (rGO) in a single electrode so that energy can be stored through both mechanisms. For example, in case of SP@rGO@Ni hybrid electrode, the rGO is charged by electrostatic force in the initial state until the electrode potential attains the redox reaction potential of the Ni(OH)_2_ till q_1_ as seen in Fig 10(b). Then the Ni(OH)_2_ is charged by the redox reaction while maintaining the redox reaction potential of the hybrid electrode, until the component reaches the full-charge state (Q_F_). After the full charge of the Ni(OH)_2_, the rGO is charged again (q_2_) to reach the maximum potential of hybrid electrode storing the charge on the surface. Hence the total charge stored in the hybrid electrode is due to both components ((q_1_ + q_2_) + Q_F_). In addition to this the maximum working potential window for the entire system (ΔV_max_) will be the sum of potentials across rGO electrode (ΔV_NF_) and hybrid electrode (ΔV_Hy_). Thus the total amount of energy stored in this hybrid system is the sum of energy stored in rGO electrode (E_NF_) and that of the hybrid electrode (E_Hy_). The hybrid electrode system has advantage at high power density due to the structural characteristics of the system compared to those of typical asymmetric capacitors^41^. In effect, the rGO electrode, which can store electrochemical energy by electrostatic force, enhances the electron transfer to the Ni(OH)_2_ and Co(OH)_2_ in the hybrid electrode system, causing a better charge transfer reaction at a high rate. To show high energy density of hybrid device for example SP@rGO//SP@rGO@Ni, the device was charged to light an LED as a demonstration for real application (Fig. 10(c–e)). After a quick charge of around 20 seconds the cell can light one LED for ~5 min. This result further demonstrates the successful utility of hybrid devices with aqueous electrolyte to realize high performance supercapacitors.

The superior electrochemical performance of the fabricated hybrid devices (SP@rGO//SP@rGO@Ni and SP@rGO//SP@rGO@Ni) can be reasonably attributed to the synergistic contribution of both materials (hybrid electrode) as well as that between the positive and negative electrodes in single device. The energy density of the hybrid devices is significantly improved because of the extended wide operation voltage window. On the other hand, the rGO in both electrodes demonstrate their distinctive features such as excellent electrochemical stability and superior conductivity due to the fact that rGO not only acts as the support for the Ni(OH)_2_ nanosheets and Co(OH)_2_ nanoflakes, but also maintains the mechanical integrity and high electrical conductivity of the overall electrode. Additionally, 3D macroporous framework as the negative electrode facilitates the transport of electrolyte ions and provides a larger surface area for charge-transfer reactions, ensuring high power density and excellent rate performance. Thus, pairing up SP@rGO@Ni or SP@rGO@Co and SP@rGO hybrid materials for hybrid devices represents a new approach to high-performance energy storage devices. Our future research will be focused on the use of organic and ionic liquid based electrolytes for such hybrid-electrode based hybrid devices.

With our earnest efforts, we have designed stunning hybrid materials based on sponge-supported rGO and transition metal hydroxides for hybrid energy storage devices. By taking into account, the processing and fabrication cost of hybrid materials and hybrid devices, inexpensive and scalable ‘dip and dry’ and CBD methods with aqueous electrolyte system have been utilized. The synergic combination of non-faradic (rGO) and redox (Ni(OH)_2_ and Co(OH)_2_) materials provides dual charge-storing mechanisms whereas 3D porous framework of sponge support allows good accessibility of electrolyte to hybrid electrodes resulting in excellent electrochemical performance. Moreover, hybrid devices using SP@rGO and SP@rGO@Ni or SP@rGO@Co as the negative and positive electrodes, respectively have been demonstrated as a bolstering approach. The proper hybridization of rGO and Ni(OH)_2_/Co(OH)_2_ improves interfacial contacts which delivers high energy as well as sustains the volume expansion during the long term charging/discharging reactions in an extended operating voltage. These encouraging findings open up the possibility of hybrid electrodes for numerous applications in hybrid energy storage devices to meet the diverse demands where high power and energy storage systems are required.

## Methods

### Fabrication of sponge@rGO (SP@rGO) composite

At first, rGO was prepared by the modified Hummer's method (supporting information S1) reported elsewhere^14^. Briefly, rGO ink with concentration of 2 mg/ml in 10 mg/ml of sodium dodecylbenzenesulfonate (SDBS) water solution was prepared by applying a 30 min bath sonication. A piece of commercially available macroporous cellulose sponge previously cleaned with water and ethanol several times was dipped into this rGO ink. The SP@rGO was washed with water to get rid of the surfactant after drying in an electrical oven for 2 h. Then the surfactant-free SP@rGO was put in a vacuum oven for another 3 h to remove the water completely.

### Development of SP@rGO@Ni and SP@rGO@Co hybrid electrodes

Subsequent depositions of nanostructured Ni(OH)_2_ and Co(OH)_2_ on to SP@rGO support substrate were carried out by simple chemical bath deposition (CBD) method. Briefly, solutions of 0.1 M NiSO_4_ and 0.1 M CoCl_2_ complexed with ammonia solution and 0.1 M hexamethylenetetramine (HMT) were prepared in de-ionized water separately in order to synthesize Ni(OH)_2_ and Co(OH)_2_ thin films, respectively. Further the pieces of SP@rGO support substrates were immersed in these baths and the bath was heated. When the bath attained the temperature of 343 K, the precipitation started in the bath. During the precipitation, a heterogeneous reaction occurs and nanostructured Ni(OH)_2_ and Co(OH)_2_ thin coatings were deposited on the SP@rGO substrates. The deposition time for both materials placed at 343 K was kept constant at 4 h. At the end, SP@rGO coated with Ni(OH)_2_ and Co(OH)_2_ are referred to as SP@rGO@Ni and SP@rGO@Co, respectively.

### Materials characterization

The X-ray diffraction (XRD) of samples was carried out using a Bruker AXS D8 Advance diffractometer with copper radiation (K_α_ of λ = 1.54 Å). The X-ray photoelectron spectra (XPS) data were obtained by X-ray photoelectron spectroscopy (XPS, Perkin-Elmer model PHI 1600). The microstructures of the samples were investigated by scanning electron microscope (SEM, Nova NanoSEM 200). Transmission electron microscopy observations were conducted using JEOL JEM-2100 operated at 200 kV. N_2_ adsorption/desorption was determined by Brunauer-Emmett-Teller (BET) measurements using Micromeritics instrument.

### Supercapacitors cell fabrication and electrochemical characterization

Two identical pieces of hybrid electrode (SP@rGO@Ni and SP@rGO@Co) each with an area of 1 cm^2^ were sandwiched by porous polypropylene film separator soaked with 2 M KOH electrolyte and assembled into a coin cell. The mass of the active materials was calculated by the mass difference method. Hybrid devices were assembled with hybrid electrode (SP@rGO@Ni and SP@rGO@Co) as the positive electrode (cathode) and SP@rGO as the negative electrode (anode). The electrodes were separated by polypropylene film separator soaked with 2 M KOH electrolyte and assembled into a coin cell. All electrochemical characteristics were evaluated by cyclic voltammetry (CV) and galvanostatic charge/discharge (CD) measurements on IviumStat and Atlas-0961 multichannel battery interface.

## Author Contributions

D.P.D. and P.G.R. designed the experiments, analyzed the data and wrote the manuscript. D.P.D. carried out synthesis and characterization of hybrid thin films. D.P.D. and R.H. designed and carried out electrochemical measurements. To the preparation and reviewing manuscript, all authors contributed equally.

## Supplementary Material

Supplementary Information Supporting Information
