# Supplementary material for: Development of hybrid materials based on sponge supported reduced graphene oxide and transition metal hydroxides for hybrid energy storage devices
Source: Sci Rep. 2014 Dec 8;4:7349. doi: 10.1038/srep07349 (PMC4258650; doi:10.1038/srep07349)
Supplement: Supplementary Information — Supporting Information [file srep07349-s1.doc]

Supporting Information

Development of hybrid materials based on sponge supported reduced graphene oxide and transition metal hydroxides for hybrid energy storage devices

*Deepak P. Dubal^a,b^*, Rudolf Holze^b^, Pedro Gomez-Romero^a,c^**

^a^Catalan Institute of Nanoscience and Nanotechnology, CIN2, ICN2 (CSIC-ICN), Campus UAB, E-08193 Bellaterra, Barcelona, Spain

^b^Technische Universität Chemnitz, Institut für Chemie, AG Elektrochemie, D-09107 Chemnitz, Germany

^c^MATGAS Research Center, Campus UAB, 08193 Bellaterra, Barcelona, Spain

**Supporting information S1**

**Synthesis of graphene oxide**

2.5 g of graphite powder, 1.25 g of NaNO_3_ and 60 mL of H_2_SO_4_ were mixed together with stirring for 30 min and cooled in an ice bath. 7.5 g of potassium permanganate was added slowly to this solution with continuous stirring overnight. Subsequently this solution was diluted with 100 mL of distilled water at high speed stirring. The temperature rapidly increased to 98 °C and maintained here for 5 h. Then, 25 mL of 30 % H_2_O_2_ were added to this solution. The black graphite suspension was converted into a bright yellow graphite oxide solution. The final prod­uct was washed several times with distilled water and HCl and then re-suspended in distilled water. The aqueous graphite oxide solution was sonicated vigorously for 2 h to exfoliate the stacked graphite oxide sheets into monolayer or multi-layered graphene oxide sheets. The con­centration of prepared graphene oxide solution is about 2 mg/ml. The rGO were synthesized by hydrothermal method. 50 ml of graphene oxide solution above solution was taken into a Teflon-lined stainless steel autoclave and heated at 180 °C for 5 h. During the heating process, graphene oxide will be reduced to rGO sheets.

**Supporting information S2**


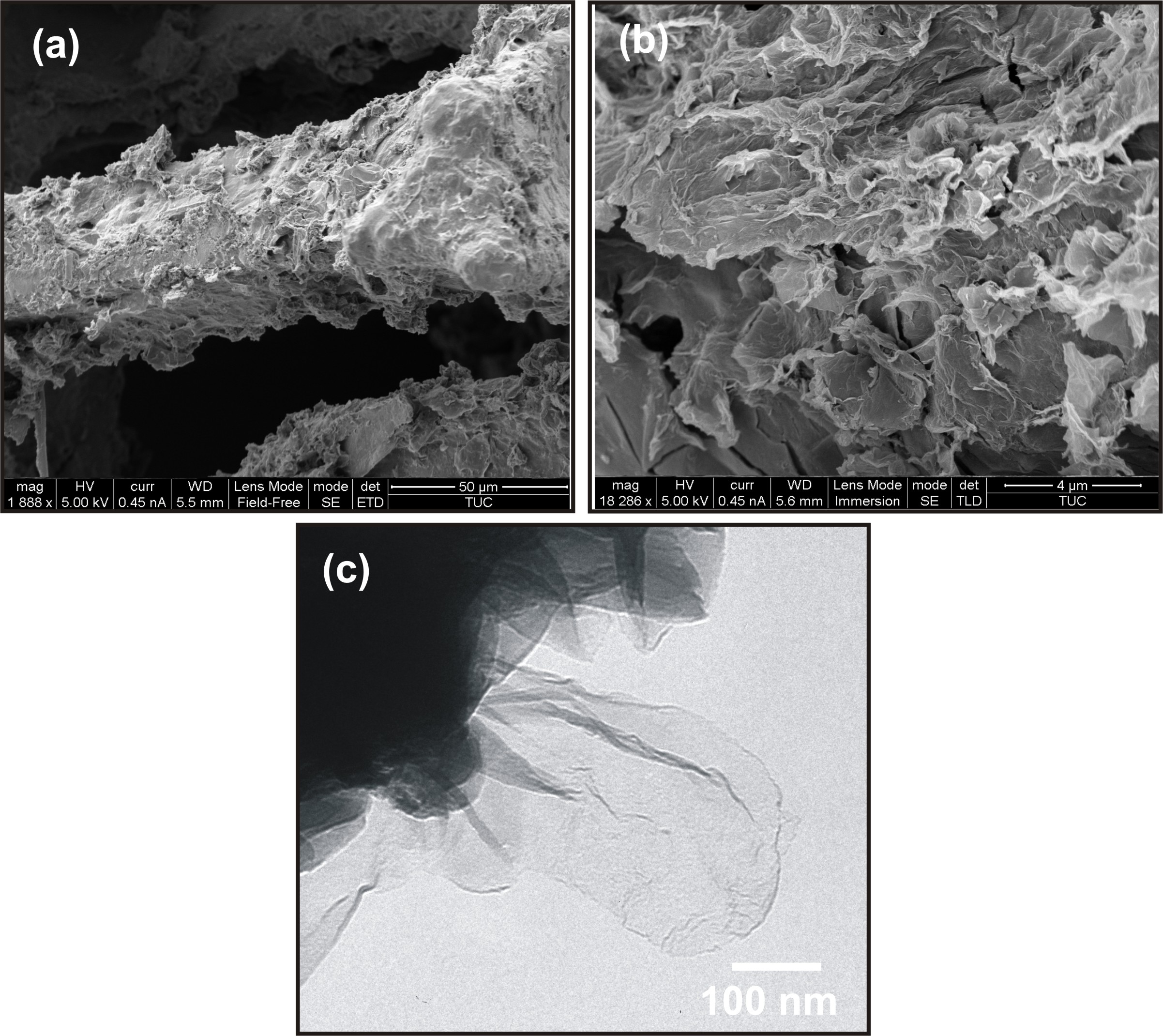


Figure S2 (a, b) SEM images of rGO coated sponge support at two different magnifications, respectively with corresponding TEM image of rGO nanosheet

**Supporting information S3**


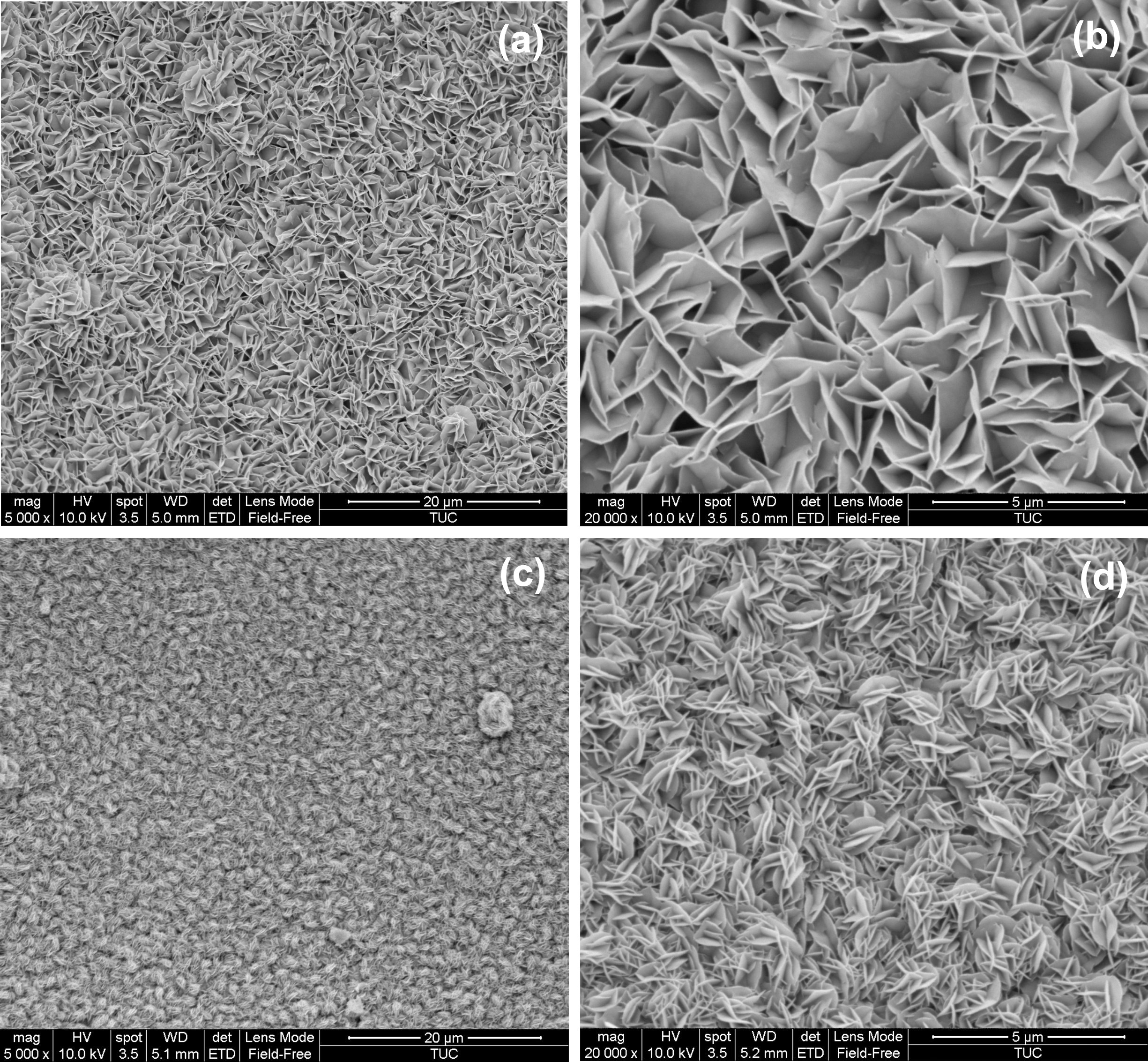


Figure S3 SEM images of (a, b) Ni(OH)_2_ and (c, d) Co(OH)_2_ on SP@rGO coated substrate at two different magnifications, respectively
